# Early Therapeutic Interventions for Newly Diagnosed Glioblastoma: Rationale and Review of the Literature

**DOI:** 10.1007/s11912-021-01157-0

**Published:** 2022-02-04

**Authors:** Mueez Waqar, Daniel M. Trifiletti, Catherine McBain, James O’Connor, David J. Coope, Leila Akkari, Alfredo Quinones-Hinojosa, Gerben R. Borst

**Affiliations:** 1 Department of Academic Neurological Surgery, Geoffrey Jefferson Brain Research Centre, Salford Royal Foundation Trust, Manchester, UK; 2 Division of Cancer Sciences, School of Medical Sciences, Faculty of Biology, Medicine and Health and Manchester Cancer Research Centre, University of Manchester, Manchester, UK; 3 Department of Radiation Oncology, Mayo Clinic Florida, 4500 San Pablo Road S, Mayo 1N, Jacksonville, FL 32224 USA; 4 Department of Neurological Surgery, Mayo Clinic, Jacksonville, FL USA; 5 Department of Radiotherapy Related Research, The Christie NHS Foundation Trust, Dept 58, Floor 2a, Room 21-2-13, Wilmslow Road, Manchester, M20 4BX UK; 6 Division of Tumour Biology and Immunology, The Netherlands Cancer Institute, Oncode Institute, Amsterdam, The Netherlands

**Keywords:** Glioblastoma, Radiotherapy, Intraoperative radiotherapy, Radiation, Brachytherapy, Neoadjuvant, Neurosurgery, Preoperative, Progression, Stem cells, Gliadel, Immunotherapy, Radiosurgery

## Abstract

**Purpose of Review:**

Glioblastoma is the commonest primary brain cancer in adults whose outcomes are amongst the worst of any cancer. The current treatment pathway comprises surgery and postoperative chemoradiotherapy though unresectable diffusely infiltrative tumour cells remain untreated for several weeks post-diagnosis. Intratumoural heterogeneity combined with increased hypoxia in the postoperative tumour microenvironment potentially decreases the efficacy of adjuvant interventions and fails to prevent early postoperative regrowth, called rapid early progression (REP). In this review, we discuss the clinical implications and biological foundations of post-surgery REP. Subsequently, clinical interventions potentially targeting this phenomenon are reviewed systematically.

**Recent Findings:**

Early interventions include early systemic chemotherapy, neoadjuvant immunotherapy, local therapies delivered during surgery (including Gliadel wafers, nanoparticles and stem cell therapy) and several radiotherapy techniques. We critically appraise and compare these strategies in terms of their efficacy, toxicity, challenges and potential to prolong survival. Finally, we discuss the most promising strategies that could benefit future glioblastoma patients.

**Summary:**

There is biological rationale to suggest that early interventions could improve the outcome of glioblastoma patients and they should be investigated in future trials.

## Introduction

Glioblastoma is the commonest primary malignant brain tumour in adults [1]. The median overall survival with standard treatment, comprising surgery and postoperative chemoradiotherapy, is just 15 months [2]. Despite decades of research, the 5-year survival remains < 5% and modern treatment fails to halt local recurrence, which occurs in > 80% of patients within 2 cm of the original surgical cavity [3, 4].

In the time between surgery and radiotherapy, remnant tumour cells remain untreated causing rapid early progression (REP), which is associated with a shorter survival [5–13]. This highlights the limitations of the current glioblastoma treatment pathway and the desperate need for new strategies. One approach involves intensified upfront therapy, which could provide timely treatment to a favourable tumour microenvironment, to counter mechanisms leading to REP and improve patient outcome. Importantly, this approach is different to simply earlier commencement of standard postoperative chemoradiotherapy, which could have a negative effect on outcome [14–16].

This review will explore the biological rationale and clinical landscape of early interventions in newly diagnosed glioblastoma, based mostly on preclinical and early phase clinical trial data. Our aim is to stimulate novel treatment approaches to improve the outcome of this deadly disease.

## Biological Justifications

**Fig. 1:**
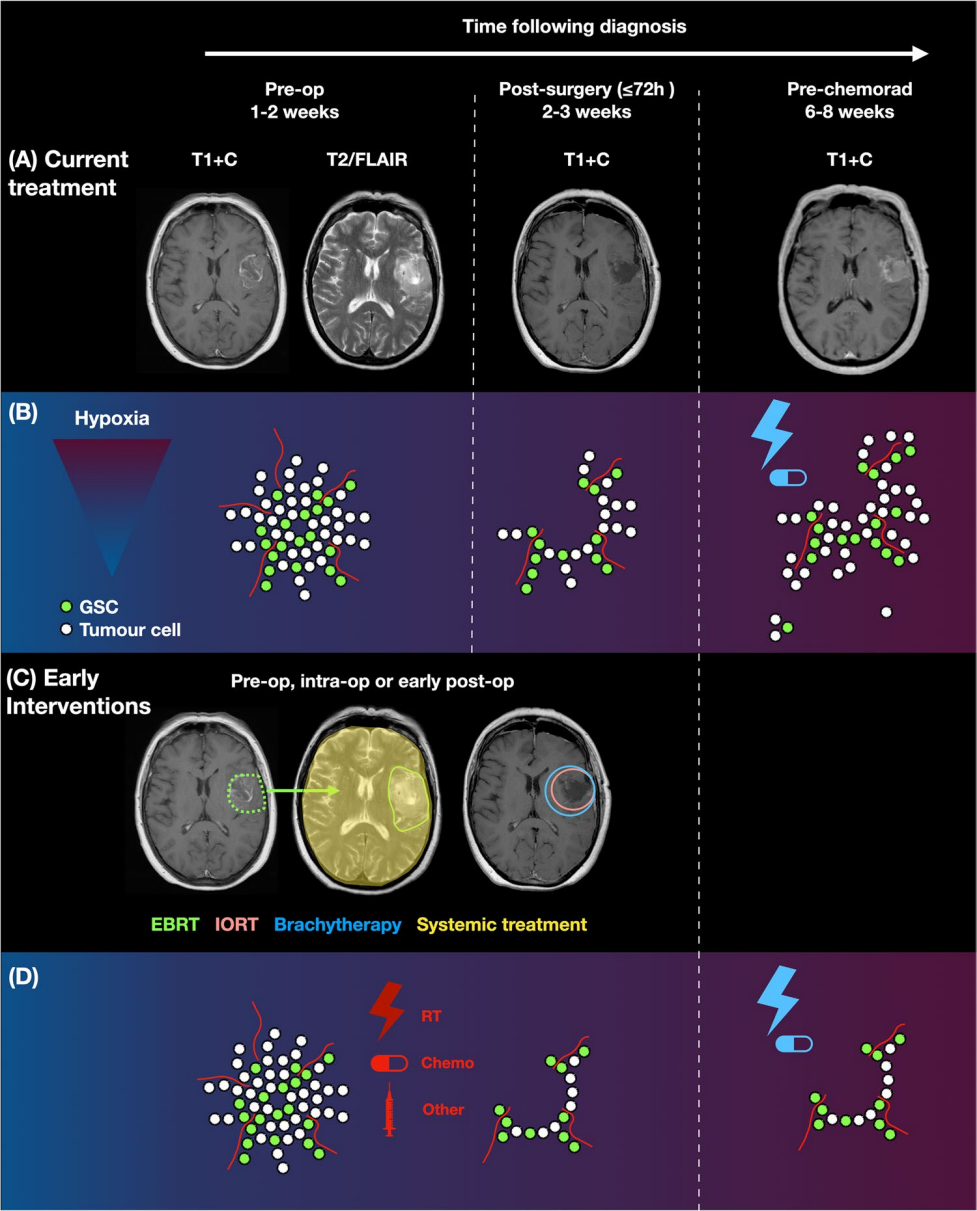
Biological rationale for early therapeutic interventions in newly diagnosed glioblastoma. **A** Limitations of the current treatment pathway for glioblastoma: serial MRI scans of a 63-year-old male who presented with seizures and dysphasia and was diagnosed with a glioblastoma. Scans are displayed preoperatively, postoperatively and pre-chemoradiotherapy demonstrating the development of rapid early progression (REP) in the time interval between surgery and postoperative chemoradiotherapy. **B** Cartoon representation of tumour cells through the current treatment pathway. Cells in the invasive margin of glioblastoma remain untreated for several weeks and may contribute to REP, potentiated by the negative biological effects of surgery. The current treatment pathway fails to prevent REP and adjuvant treatment is also delivered to a relatively more hypoxic postoperative tumour bed. **C** Sites of action of early therapeutic interventions including radiotherapy and systemic therapies. **D** Cartoon representation of the beneficial effects of early interventions on tumour cells, leading to fewer tumour cells with time, through earlier treatment of the invasive margin and subsequent prevention of REP. Early interventions also act on a relatively less hypoxic microenvironment and could increase the effectiveness of chemoradiotherapy. Abbreviations: T1 + C, T1 with contrast; Pre-op, preoperative; GSC, glioma stem cell; chemorad, chemoradiotherapy; EBRT, external beam radiation therapy; IORT, intraoperative radiotherapy; RT, radiotherapy; chemo, chemotherapy

There are several biological advantages to earlier interventions (Fig. 1).

### Rapid Early Progression

Approximately half of all glioblastoma patients develop macroscopically observed REP between surgery and postoperative radiotherapy, which is associated with a shorter survival (Table 1 and Fig. 1A) [5–13]. From a biological perspective, macroscopically observed REP is associated with extent of resection and volume of residual disease and more frequently observed in patients with subtotal resection [6, 8, 10]. In addition, preclinical studies suggest that surgery potentiates the proliferative and migratory state of glioblastoma cells on a microscopic level [17, 18]. In one clinical study of patients with multifocal glioblastoma where just one tumour was biopsied, the biopsied tumour grew faster than the non-biopsied tumour, with an increase in tumour cell motility, migration and proliferation [17]. The poorer outcomes of patients after subtotal resection could be in part explained by REP [19].

**Table 1:**
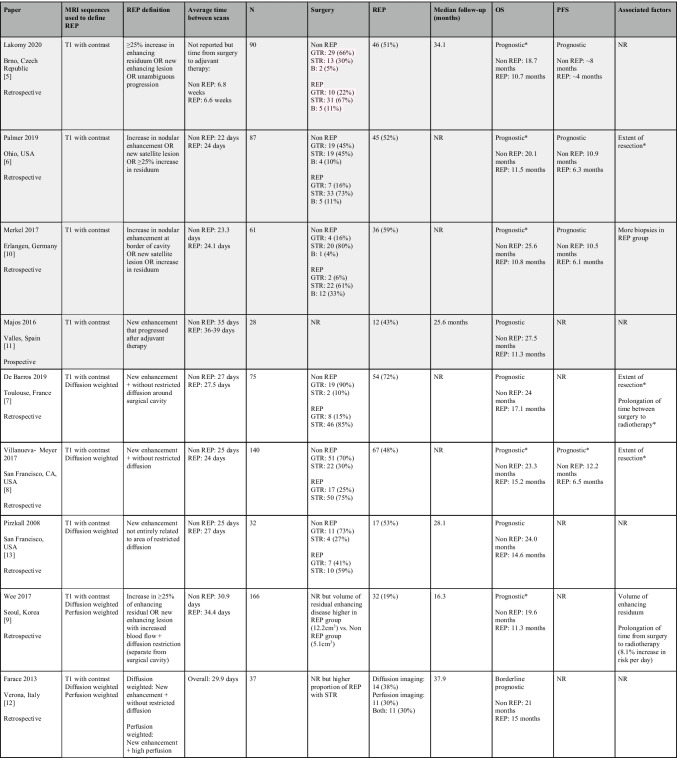
Rapid early progression (REP) in glioblastoma. Studies have been grouped into two groups: those using just T1 contrast enhancement as an indicator of REP (shaded light grey) and those using T1 contrast enhancement in combination with diffusion and/or perfusion weighted imaging (shaded white)

^*^Indicates significance in multivariate analysis. Abbreviations: *GTR*, gross total resection; *STR*, subtotal resection; *B*, biopsy; *REP*, rapid early progression; *OS*, overall survival; *PFS*, progression-free survival; *NR*, not reported

### Hypoxia

Neoadjuvant treatment may better target tumours due to the higher preoperative tumoural blood flow and less hypoxia compared to postoperative residual tumours [20]. Magnetic resonance imaging (MRI) studies have demonstrated the ischaemic side effects of surgery, which are correlated with more aggressive recurrence patterns. Indeed, postoperative diffusion-weighted MRI demonstrates areas of restricted diffusion indicative of ischaemia, in up to 90% of patients and more frequently after gross total resection (GTR) [21]. Although GTR improves outcome, ischaemic lesions are independently associated with a shorter survival and multifocal tumour recurrence [22]. In addition, hypoxia is associated with treatment resistance and is independently related to worse outcomes for glioblastoma patients [23, 24]. Hypoxia alters the glioblastoma microenvironment towards a more aggressive phenotype largely through increased hypoxia-inducible-factor (HIF) signalling. These transcription factors have several effects including: maintenance of cancer stem cell stemness and promotion of tumour cell dedifferentiation [25, 26]; upregulation of vascular endothelial growth factor (VEGF) mediated angiogenesis [26]; increased chemokine signalling to promote vasculogenesis [26]; upregulation of microRNAs involved in resistance to temozolomide [27]; promotion of a proneural-to-mesenchymal transition [28]; and a shift towards a growth promoting metabolome with increased rates of glycolysis [28]. Treatment of a biological system containing fewer aggressive hypoxic regions may therefore improve the overall outcome.

### Untreated Tumour Cells

Glioblastoma is a diffusely infiltrative disease whose microscopic margins extend beyond those visible on modern imaging or intraoperative appearance [29]. Unresected remnant cells are exposed to surgery induced tumour potentiating factors. In addition, these cells are not treated for at least 4–6 weeks, the average time period between surgery and postoperative chemoradiotherapy [14]. This time period is longer than the predicted doubling time of the disease [30]. Furthermore, temporal heterogeneity of glioblastoma increases with time, with an increase in overall mutation burden and subclones after completion of treatment [31]. Targeting an earlier biological tumour system with less heterogeneity may therefore translate into clinical benefit.

In summary, these observations suggest a role for earlier interventions that could include traditional modalities like surgery, radiotherapy and chemotherapy, and novel approaches including targeted agents such as immunotherapy. The following sections will provide an overview of these strategies.

## Clinical Interventions

### Surgery

Early repeat surgery could be used as a treatment for REP. It is currently performed for unintended residual disease, though done very rarely given the widespread use of tools to aid maximal safe resection. When employed, outcomes appear to be favourable, though length of stay is increased [32, 33]. However, early repeat surgery may not be technically possible and/or considered too high risk in most cases because of the location of the remnant/progressive tumour [5, 10]. This surgical risk assessment is crucial, as demonstrated in a large UK national study of residual enhancing disease in glioblastoma. Although 44/80 patients had residual enhancing disease, none underwent further surgery [34]. Additional arguments against surgery include the fact it may delay postoperative therapy further for untreated cells and does not exploit the favourable early tumour microenvironment.

### Systemic Treatment

#### Temozolomide

Systemic treatments could exploit and target the biological justifications for early interventions. Careful consideration is required as to the exact time point of use, which could be preoperatively or in the early postoperative period. In reference to the latter, chemotherapy could in theory result in immunosuppression and wound healing difficulties. However, this was not demonstrated in a recent case–control study of temozolomide use within 7 days after surgery, followed by the Stupp protocol (6 weeks radiotherapy and concurrent temozolomide). Indeed, a significantly longer survival was noted in the early temozolomide group (23.0 months vs. 17.0 months), with no significant increase in complications [35]. These findings require validation, though early temozolomide may be of specific benefit to patients with O6-methylguanine-DNA methyltransferase (MGMT) promotor methylation. Future research should also explore the increased survival noted in REP patients with MGMT promotor methylation versus those with unmethylated promotors, to test whether early temozolomide use could counter REP cellular processes [6].

#### Immunotherapy

Neoadjuvant immunotherapy is of great interest for newly diagnosed glioblastoma (Table 2) after encouraging preliminary results in recurrent glioblastoma [36]. Cloughesy et al. found neoadjuvant immunotherapy to be superior to postoperative immunotherapy alone in recurrent glioblastoma. Thirty-five patients were randomised to neoadjuvant/postoperative anti-programmed death ligand 1 (anti PD-1) or postoperative anti-PD1 alone, prior to re-resection. The median survival was almost doubled with neoadjuvant treatment (13.7 vs. 7.5 months) [36]. Neoadjuvant treatment upregulated the expression of genes related to key immune pathways such as interferon gamma responsiveness to a greater extent compared to standard postoperative therapy. This effect may translate to patients with newly diagnosed glioblastoma and data is awaited from an ongoing trial (NCT04583020) [37]. Preliminary experience of 3 patients treated with neoadjuvant anti PD-1 is encouraging (2 out of 3 patients with newly diagnosed glioblastoma survived ≥ 28 months) [38].

**Table 2:** Clinical trials of early time point interventions for glioblastoma that are currently recruiting or soon to begin. *POBIG stands for PreOperative Brain Irradiation in Glioblastoma—an upcoming phase I dose escalation trial of neoadjuvant radiotherapy for newly diagnosed glioblastoma at the senior author’s institution. Abbreviations: *BET*, bromodomain and extra-terminal domain; *PD-1*, programmed death ligand 1; *CDK*, cyclin dependent kinase; *CTLA-4*, cytotoxic

Reference	Phase	Patients	Trial intervention (see legend)	Time period	Other treatment
NCT03582514 (POBIG*) Manchester, UK	I	Newly diagnosed	Radiotherapy	Neoadjuvant	None
NCT05074992 London, UK	II	Newly diagnosed	Ipilimumab	Neoadjuvant	Not specified
NCT03576612 Multi-centre USA	I	Newly diagnosed	Aglatimagene besadenovec (AdV-tk, gene therapy) injected into wall of surgical cavity	Intraoperative	Early postoperative valacyclovir and nivolumab
NCT02685605 INTRAGO-II International multi-centre	III	Newly diagnosed	Intraoperative radiotherapy (Intrabeam device)	Intraoperative	Not specified
NCT03055208 Mannheim, Germany	II	Newly diagnosed	Gamma knife radiosurgery	Early postoperative (24–72 h)	Not specified
NCT04583020 Beijing, China	II	Newly diagnosed suitable for surgical resection	Camrelizumab (anti PD-1)	Neoadjuvant + adjuvant	Surgical resection 60 Gy radiotherapy Temozolomide
NCT04209790 PA, USA	II	Newly diagnosed suitable for surgical resection	Radiotherapy Temozolomide	Neoadjuvant	Not specified
NCT04047303 Multi-centre USA	I/II	Recurrent gliomas suitable for salvage surgical resection	CC-90010 (BET protein inhibitor)	Neoadjuvant	Not specified
NCT04888611 Shanghai, China	II	Recurrent suitable for resection	Camrelizumab (anti PD-1) and dendritic cell vaccine (or placebo)	Neoadjuvant + adjuvant	None
NCT02133183 Multi-centre USA	I	Recurrent suitable for resection	Sapanisertib (mTOR inhibitor)	Neoadjuvant + adjuvant	Not specified
NCT04606316 Boston, USA	I	Recurrent suitable for resection	Nivolumab (anti PD-1) ± ipilimumab (anti CTLA-4)	Neoadjuvant	Not specified
NCT04323046 San Francisco, USA	I	Recurrent suitable for resection	Nivolumab (anti PD-1) ± ipilimumab (anti CTLA-4)	Neoadjuvant	Not specified
NCT03834740 Multi-centre USA	0/II	Recurrent suitable for resection with suitable mutation (e.g. Rb, CDKN2A, mTOR +)	Ribociclib (CDK4/6 inhibitor) and everolimus (mTOR inhibitor)	Neoadjuvant	Not specified
NCT02933736 Barrow, USA	I	Recurrent suitable for resection, with retinoblastoma positivity	Ribociclib (CDK4/6 inhibitor)	Neoadjuvant	Not specified

Use of neoadjuvant immunotherapy requires careful consideration of other treatments employed in this time period that can have immunomodulatory effects. Steroids are one example that can negate the potentiating effects of surgery [17]. However, steroids are not by themselves cytotoxic and may have offset the survival benefit of postoperative immunotherapy in the CheckMate 143 trial [39]. Such concerns have led to reluctance to use high-dose steroids alongside immunotherapy.

### Local Therapies at the Time of Surgery

#### Gliadel Wafers

Local intraoperative therapies can overcome the drug constraints of the blood–brain-barrier. Gliadel wafers (carmustine in a biodegradable polymer—providing the alternative name of carmustine wafers) deliver local chemotherapy over a period of 5–7 days, with degradation of the polymer occurring over 5–6 weeks [40]. The only completed randomised trial to evaluate their efficacy is from the pre-temozolomide era and although a marginal improvement in overall survival was noted in the Gliadel group, this was not statistically significant during long-term follow-up [41, 42]. There are also concerns about the toxicity of this treatment with respect to high seizure rates and wound complications, though these are not universally reported, and were comparable to non-Gliadel patients in the aforementioned trial and large centre experiences [42–44]. This controversy has limited the eligibility of patients with Gliadel to participate in future trials [45]. The combined efficacy of Gliadel and temozolomide is currently being evaluating in an ongoing randomised trial [46]. Other intraoperative strategies such as direct intratumoural injection of carmustine and local immunotherapy have achieved disappointing clinical results [47, 48].

#### Nanoparticles and Stem Cell Therapy

There is significant preclinical interest in the use of nanotechnology and human stem cells, to enhance delivery of anti-tumour therapies and target specific tumour cells. Nanoparticles can be conjugated to anti-tumour molecules and enhance drug delivery [49]. For example, the topoisomerase inhibitor camptothecin was conjugated to a nanoparticle hydrogel self-assembly drug system and injected into the surgical cavity of a mouse glioblastoma model after resection. The median survival of mice treated with nanoparticles was almost doubled [50]. In addition to mere vehicles, nanotechnology can encompass biocargoes including small interfering ribonucleic acids (siRNAs) and DNA altering technology (e.g. CRISPRs/Cas9), to directly target specific molecular alterations [51]. In this way, intratumoural injection of liposomes containing interferon-beta in five high-grade gliomas achieved an encouraging overall survival of 17 months [52].

Human stem cells show tropism to brain pathology such as tumours and can cross the blood–brain-barrier, offering another vehicle system. They can be delivered systemically or locally. Human adipose derived mesenchymal stem cells (hAMSCs) are of particular interest as, compared to embryonic stem cells, they are derived from readily available adipose tissue, are more genetically stable and have a lower senescence ratio [53]. Preclinical experience is promising, particularly when combined with nanotechnology, to avoid potential immunogenicity that can accompany viral transfection [54].

#### Viral and Vector Mediated Therapy

Naturally occurring viruses such as adenoviruses and herpes simplex viruses can be genetically engineered to target glioblastoma cellular proteins and exert anti-tumourigenic effects when administered systemically or locally. The resulting cytotoxicity and cell lysis (‘oncolysis’) can induce an immune response, further strengthening the overall effect [55]. Early-phase studies evaluating oncolytic viral therapy delivered to the surgical bed for recurrent glioblastoma have reported a high rate of adverse events (39–67%), so the technique requires further evaluation [55]. An alternative approach is called gene-mediated cytotoxic immunotherapy—using locally delivered viral vectors in combination with anti-viral agents and immune-modulating agents, to stimulate a systemic vaccine effect [56]. Aglatimagene besadenovec (AdV-tk) is an adenoviral vector expressing the herpes simplex virus thymidine kinase, which is currently being evaluated in this way for newly diagnosed glioblastoma (NCT03576612) [57].

### Radiotherapy

#### Dose and Dose Escalation

Postoperative radiotherapy utilises modern external beam radiation therapy (EBRT) techniques such as intensity modulated radiation therapy (IMRT) and volumetric modulated arc therapy (VMAT), which increase dose conformity. Nonelderly patients receive 60 Gy, though doses up to 75 Gy are well tolerated [58]. However, most contemporary trials of dose escalation have found no improvement in outcome when doses above 60 Gy are given at the conventional postoperative time point [59–64]. Alternative strategies are therefore required and the following sections will review those with clinical results. Notably, FLASH radiotherapy, involving instantaneous high-dose radiation therapy, has not yet made it to clinical testing although preclinical results are promising [65].

#### Intraoperative Radiotherapy

Intraoperative radiotherapy (IORT) using photons or electrons is administered after maximal resection. Modern IORT for gliomas uses Intrabeam (Carl Zeiss®), a mobile device that delivers low-energy photons (30–50 kV), thus alleviating the need for operating room radiation shielding [66]. Treatment is delivered through spherical applicators depending on the size of the surgical cavity [66].

In a recent phase II trial—INTRAGO (INTRAoperative radiotherapy for GliOblastoma), 15 patients with newly diagnosed glioblastoma were treated with a median IORT dose of 25 Gy. Encouraging results were obtained with 2/15 cases of local progression and a median overall survival of 17.8 months. The two cases of local progression included one patient that received the lowest dose of radiation at 20 Gy and one who could not receive postoperative concurrent chemoradiotherapy. Radiation necrosis (*n* = 5) occurred at each dose of radiation in roughly equal numbers [67]. These results were affirmed by other centres reporting a median OS of around 18 months with IORT for glioblastoma [68, 69]. A future phase III trial (INTRAGO II) will test the efficacy of intrabean based IORT (NCT02685605) [70].

#### Brachytherapy

Brachytherapy involves administration of radioactive isotopes into the tumour or surgical cavity that decay with time, releasing radiation to surrounding tissue. In glioblastoma, brachytherapy has been most commonly used with iodine-125 (I-125), though two phase III randomised trials did not find that it improved survival [71–73].

Recent studies have reported favourable outcomes with brachytherapy in the temozolomide era [73]. Furthermore, advances in brachytheraphy technology have improved its safety, leading to more support for its use [74]. New brachytherapy systems such as the GammaTile® include absorbable radioisotope carrier systems embedding caesium-131 (Cs-131) seeds. These do not require surgical removal and they prevent direct contact between radioactive seeds and the brain surface. From an efficacy perspective, Cs-131 has a shorter half-life than radioisotopes such as I-125, providing a cumulative radiation boost earlier than I-125 [74]. Early experience in brain metastases has demonstrated excellent local control of 100% [75, 76]. The rates of radiation necrosis with Cs-131 also appear lower than I-125, reported at 0–11% [76, 77]. Studies evaluating GammaTile® for glioblastoma are ongoing (NCT04427384) [78].

#### External Beam Radiation Therapy

Use of EBRT has two potential time points of use—(1) in the early postoperative stage or (2) preoperatively.

### *Early (*≤ *3 Weeks) Postoperative External Beam Radiation Therapy*

Earlier commencement of standard postoperative chemoradiotherapy does not show survival benefit [14, 15]. Some studies have evaluated early postoperative SRS (≤ 3 weeks) that is additional to standard chemoradiotherapy. Smith et al. reported a phase I/II trial Gamma-Knife SRS delivered ≤ 2 weeks postoperatively (including Gliadel wafers). Thirty patients were included and the median overall survival was < 12 months overall [79]. In contrast to these disappointing results, Duma et al. described a favourable overall survival of 23 months in 174 patients using postoperative Gamma-Knife SRS to the FLAIR abnormality. Treatment was given a median of 18 days postoperatively. Thus, early postoperative EBRT in the form of SRS has not demonstrated conclusive efficacy. A future trial will test SRS ≤ 48 h postoperatively for residual tumour [80].

### Preoperative External Beam Radiation Therapy

Preoperative radiotherapy for brain metastases is safe [81] but has only been historically tested in glioblastoma patients with whole brain radiotherapy (WBRT) as described by Seiler et al. in 1979 [82]. In 10 patients with new suspected glioblastomas, 30–40 Gy WBRT over 3–4 weeks was given preoperatively, followed by a 2–3-week delay to surgery with subsequent postoperative WBRT of 25–40 Gy in 3–4 weeks. Although this is an outdated and abandoned technique, a relatively favourable median survival of 12 months was achieved in this pre-temozolomide era experience [82]. Preoperative radiotherapy is an interesting strategy to explore in the modern era using contemporary radiotherapy techniques of IMRT/VMAT.

Preoperative chemoradiation is currently being trialled prospectively (NCT04209790) in biopsy confirmed glioblastoma patients [83]. Prior tissue confirmation of histological or molecular diagnosis has the potential of delaying the treatment, morbidity and mortality [84]. The possibility of misdiagnosis is rare using modern MRI techniques, that have a high sensitivity, but the effects of EBRT on prognostic molecular mutations in glioblastoma is unclear and will require further evaluation [85, 86].

## Discussion

Early interventions have advantages and disadvantages (Table 3 and Fig. 1C). Their common advantage is the potential to target tumour cells that are otherwise only treated 6–8 weeks after diagnosis, whilst stimulated to become more biologically aggressive from surgery. These cells are potentiated by the post-surgical inflammatory response that stimulates wound repair. Patient factors are also an important consideration as not all patients may be suited to each technique. For example, early postoperative therapies suit patients presenting acutely unwell requiring urgent surgical intervention.

**Table 3:** Early therapeutic interventions for newly diagnosed glioblastoma: advantages and disadvantages of different interventions

	Early therapeutic interventions for newly diagnosed glioblastoma: an evaluation of different interventions					
	Second surgery	Systemic agents	IORT	Brachytherapy	Pre-op EBRT	Early post-op EBRT
**Advantages**	• Can allow definitive removal of unintended residual disease that itself is associated with REP	• Neoadjuvant immunotherapy may be more effective than adjuvant • Temozolomide may be of benefit to patients with MGMT promotor methylation • Combination with local radiotherapy techniques is possible	• Promising results in early phase trials • Logistically easy to implement • Potentially less dose to organs at risk • High surface dose	• Potentially less dose to organs at risk • High surface dose • Modulation of spatial dose is possible to some extent	• Uses available technology that can deliver radiation with precision • Spatial dose modulation with potential to cover all disease • Accurate estimation of dose to organs at risk • Opportunity to study short-term irradiation response	• Uses existing technology that can deliver radiation with precision • More precise target and margin delineation on MRI • Accurate estimation of dose to organs at risk
**Disadvantages**	• Increased risk of complications related to surgery and anaesthesia • Could delay postoperative chemoradiotherapy • Negative biological effects of surgery	• Systemic side effects may increase risk of postoperative complications (e.g. poor wound healing, infection)	• Cost of technology • Need for training • On-site availability of radiotherapy delivery experts • Shape of surgical cavity must be appropriate • Need for intraoperative imaging • Only targets 5–10-mm margin from surgical cavity • No spatial dose modulation	• Has not demonstrated efficacy in previous randomised trials • Cost of technology • Need for training • On-site availability of radiotherapy delivery experts • Difficult to precisely calculate dose to residuum/organs at risk	• Treatment on basis of imaging diagnosis alone • Requires logistical alignments • Patients requiring urgent surgery may not be suitable • The effect of radiotherapy on clinically relevant markers is unclear	• May have ‘missed window’ to counter negative biological effects of surgery • Patients with complications of surgery may not be able to have this therapy • First attempts did not provide promising results

From a biological point of view, postoperative treatment strategies have the common disadvantage that tumour potentiating effects of surgery may have already occurred. In addition, surgery can induce stem cell like changes in peritumoural astrocytes that may not be reversible [87]. Furthermore, surgery leads to immune cell infiltration that promotes tumour cell proliferation and potentially decreases radiosensitivity [88]. In this regard, preoperative treatment given in advance of the operation theoretically renders tumour cells less aggressive at the time of surgery. Indeed, radiation induced cellular senescence in glioblastoma appears to be time dependent. Zhang et al. exposed the LN229 glioblastoma cell line to 2–8 Gy radiation and found increased cell senescence and decreased cell cycle checkpoint regulation at 7 days versus 12 hours post-irradiation [89]. From an oxygenation point of view, preoperative tissue is also more oxygenated compared to postoperative residuum, making it plausibly more sensitive to preoperative EBRT and neoadjuvant immunotherapy [23, 90].

Other biological considerations for early interventions relate to the potential of mutual synergism. Targeted agents could be used with early time point radiotherapy strategies, though clinical results using radiosensitisers and immunotherapy in the normal postoperative setting are disappointing to date [91]. Indeed, the CheckMate 498 and 548 randomised trials that evaluated nivolumab (anti-programmed death ligand-1) have failed to show an improvement in overall survival with combined immunotherapy and radiotherapy for newly diagnosed glioblastoma patients [92, 93].

Advances in nanotechnology may facilitate delivery of drugs in a more efficient way, or even as radiosensitisers in their own right, as with gold nanoparticles that can absorb ionising radiation [49]. This may be particularly effective with longer courses of radiation. Neoadjuvant immunotherapy and early time point radiotherapy strategies may be of interest as preclinical data corroborates their combined efficacy [89, 94]. However, more data is needed to understand how immunotherapy can best be combined with radiotherapy using more advanced model systems replicating the immune microenvironment of glioblastoma.

Several considerations are important for early time point radiotherapy. Current EBRT techniques allow individualised treatment plans enabling dose boosts and custom margins (i.e. dose modulation). The use of protons in particular allows dose modulation whilst increasing normal tissue sparing [95]. However, more data is required regarding the early time point irradiation response to advanced preoperative EBRT strategies. In contrast to EBRT, IORT delivers a highly spherical dose of radiation to a 5–10-mm margin, though not all surgical cavities may be suitably shaped for this technique. Brachytherapy delivers a radiation dose that is dependent on surgical implantation technique and 5–8 mm around the surgical cavity with newer GammaTile® technology [74]. Notably, IORT/brachytherapy dose distributions potentially do not reach more distally invasive glioblastoma cells. This phenomenon could explain the discrepancy seen between the local and overall progression free survival seen in the INTRAGO trial (17.8 months versus 11.3 months) [67]. Preoperative EBRT and neoadjuvant immunotherapy have challenges in that they require patient treatment based on imaging diagnosis alone to prevent additional surgical interventions. They also require consideration of the timing of surgery in particular for severely symptomatic patients.

Although there is strong biological rationale to support early interventions for newly diagnosed glioblastoma, clinical evidence is currently at an early stage and/or retrospective in design, with inherent selection bias that precludes extended analysis of outcomes. Of interest, early interventions have demonstrated benefit in other solid tumours. For example, neoadjuvant chemotherapy/radiotherapy can downstage locally advanced breast cancer, sarcoma and several gastrointestinal cancers, improving the likelihood of organ preserving gross total resection [96–99]. This does not always translate into a long-term survival benefit however, as demonstrated for retroperitoneal sarcoma [100]. As glioblastoma is a diffusely infiltrative solid tumour, total tumour removal is not possible or even the aim of therapy, but rather, extending survival, which itself has proved challenging with the current treatment pathway. Future outcome data from larger trials is awaited to evaluate the efficacy and role of early interventions for glioblastoma.

## Conclusion

There is biological rationale to suggest that early interventions could benefit the outcome of glioblastoma patients. Additional therapy at an earlier time point treats a better oxygenated tumour in a treatment naïve biological system, with less molecular heterogeneity. Neoadjuvant immunotherapy and early time point radiotherapy strategies are of specific interest as they can target invasive tumour cells that cannot be resected. Early interventions could finally lead to the long-awaited improvement in survival for glioblastoma and require further investigation.

## Data Availability

Data sharing not applicable to this article as no datasets were generated or analysed during the current study.
